# Biomimetic design of iridescent insect cuticles with tailored, self-organized cholesteric patterns

**DOI:** 10.1038/s41467-020-17884-0

**Published:** 2020-08-14

**Authors:** Adriana Scarangella, Vanessa Soldan, Michel Mitov

**Affiliations:** 1 Centre d’Elaboration de Matériaux et d’Etudes Structurales, CEMES, CNRS, Toulouse, France; 2 Centre de Biologie Intégrative, CBI, Microscopie Electronique Intégrative, METi, CNRS, University of Toulouse, Toulouse, France

**Keywords:** Biophysics, Materials for optics, Photonic crystals

## Abstract

Replicating biological patterns is promising for designing materials with multifaceted properties. Twisted cholesteric liquid crystal patterns are found in the iridescent tessellated cuticles of many insects and a few fruits. Their accurate replication is extremely difficult since discontinuous patterns and colors must coexist in a single layer without discontinuity of the structures. Here, a solution is demonstrated by addressing striped insect cuticles with a complex twisted organization. Geometric constraints are met by controlling the thermal diffusion in a cholesteric oligomer bilayer subjected to local changes in the molecular anchoring conditions. A multicriterion comparison reveals a very high level of biomimicry. Proof-of-concept prototypes of anti-counterfeiting tags are presented. The present design involves an economy of resources and a high versatility of chiral patterns unreached by the current manufacturing techniques such as metallic layer vacuum deposition, template embossing and various forms of lithography which are limited and often prohibitively expensive.

## Abstract (web summary)

Replicating biological patterns is promising for designing materials with multifaceted properties but replication of twisted cholesteric liquid crystal patterns found in insects is extremely difficult. Here, the authors use liquid crystal oligomers to reproduce the textural, structural and color properties of biological liquid crystals.

## Introduction

A huge diversity of patterns and colors is found in nature and at different length scales^1,2^, which inspire the design of materials for addressing modern challenges in bioengineering, energy capture and conversion, sensing, communication or data processing^3^. However, current biomimetic materials rarely capture the range of structural complexity observed in natural materials, as acknowledged in recent reviews^3–5^. In addition, a majority of the so-called biomimetic materials rely on manufacturing processes that have scalability limitations.

**Fig. 1:**
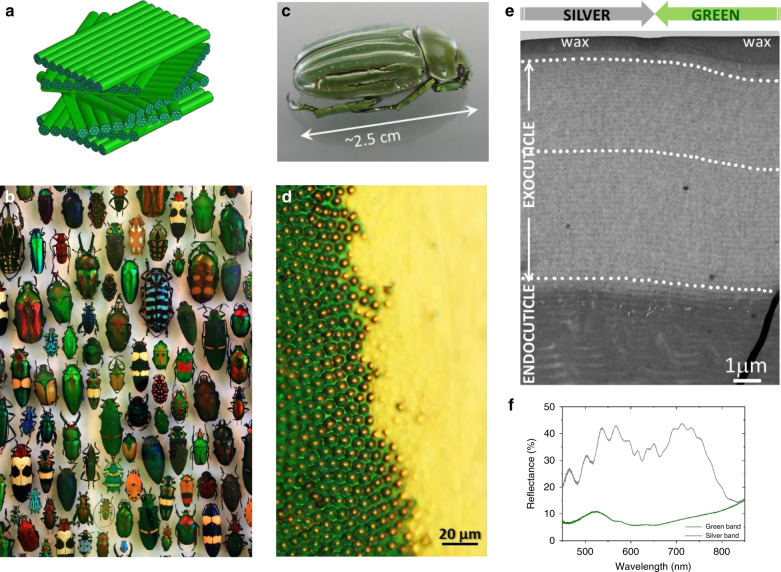
Lessons from nature. **a** In the insect cuticle, chitin macromolecules form fibrils that wrap with proteins and assemble into fibres, which assemble into bundles. The bundles arrange themselves parallel to each other and form pseudolayers. Finally, the chitin fibre-protein layers stack into a twisted plywood Bouligand structure^7^. The structure is not a discrete multilayer system; instead, a continuous twist occurs along the axis perpendicular to the fibres, which interpenetrate from one pseudolayer to the next. For this reason, the term pseudolayer is preferred over layer. A 360° rotation of the fibre orientation defines the helical pitch. (Adapted from ref. ^75^. Creative Commons license.) **b** The insect world includes a very large variety of tessellated (patterned) cuticles with bumps, pits, pixels, bands, spots or patches and a diversity of structural colors (Staatliches Museum für Naturkunde, Karlsruhe, Germany. Image by H. Zell. Creative Commons license). **c** Two-band iridescent cuticle of *Chrysina gloriosa*. **d** Green and silver bands at their interface as observed by optical microscopy (reflection mode, unpolarized light). **e** TEM images of transverse structures at the interface between green and silver bands and close to the top surface. White dots underline the stripes in the fingerprint texture. The transition between curved and straight stripes is observed to be very gentle. The last part of the cuticle consists of a wax layer^76^, which may serve to restrict water loss and prevent desiccation^77^. **f** Reflection spectra of green and silver bands.

The biomimetic design of performance materials mimicking the structure of insect cuticles is still in its infancy because the design tools necessary for the control of nano- and microscale patterns with a high level of versatility are still pending^6^. In the present work, we approached the problem by focusing on tessellated (patterned) and iridescent cuticles with a complex twisted organization of chitin fibres. The cuticles of insects have been pivotal to their successful adaptation to the external environment. Both the exocuticle and endocuticle involve twisted cholesteric liquid crystal (CLC) organization of pseudolayers of chitin macromolecules^7^ (Fig. 1a), which causes a spatially varying index of refraction. The term pseudolayers is used instead of layers because the CLC structure is not a layered system. A stack of layers with an orientational order of rod-like molecules in each plane is often drawn to represent the CLC structure. However, these layers have no physical basis and this representation is only a guide for the observer, as the rod-like molecules have no positional order. Each layer behaves like a uniaxial anisotropic medium with the slow axis parallel to the molecules and the fast axis perpendicular to them when the CLC structure is modelled as a layered system. These two axes twist regularly by a constant angle and without discontinuity from one layer to the next. When light propagates through a CLC in the Bragg regime, the medium behaves like a multilayer system, producing interferential colors. The orientation of the molecular director (the orientation of the elongated chitin fibres depicted in Fig. 1a) and thus the optical axis are modulated helicoidally, rotating in a plane that is perpendicular to the helical axis.

For circularly polarized light propagating along the helix axis with the same handedness as the helix, a CLC exhibits a Bragg reflection band over the wavelength range of *(n*_o_*–n*_e_*)p*, where *n*_o_ and *n*_e_ are the ordinary and extraordinary refractive indices and *p* is the helical pitch^8^. The central wavelength *λ*_0_ is: *λ*_0_ = *np*, where *n* = (*n*_o_ + *n*_e_)/2 is the average refractive index.

In biomimicry, many of the same species come up^9^: geckos, spiders, and butterflies. The latter are dominant in the field of photonic structures. Air layers and pillar-supported chitinous layers alternate in the twist-free, multilayer structure of butterfly wings, which leads to optical interference^10^. There are many replication methods, which are based on three concepts that use wings as templates^11^: coating, modification and filling. The morphology of the replicas can be hard to control. The choice of the method depends on the requirements of the situation.

Biomimetic materials aiming at reproducing the cuticles of beetles as closely as possible are especially rare. They have addressed the untextured, gold scarab beetle *Plusiotis resplendens*^12,13^. The samples consist in the superposition of three layers: a nematic LC layer, which acts as a half-wave retardation plate, is inserted between two left-handed cholesteric layers. The fabrication methods used a UV-crosslinked elastomer^12^ or chiral cellulose nanocrystals after water evaporation^13^. In the latter case, a non-uniformity of the reflection colors at the surface of the sample resulting from the evaporation process is reported. These three-layer systems present interfaces. When optical performance is desired, interfaces likely add parasitic reflections and light scattering to Bragg reflections. Besides, the cuticle of *P. resplendens* is interface-free: the cholesteric-nematic interfaces do not indeed exhibit any discontinuity^14^.

In contrast to nontextured reflective cuticles, tessellations as found in the cuticles of many insects^15–17^ (Fig. 1b), and a few fruits^18,19^ are challenging to duplicate via a biomimetic design. First, the pitch length and the helical axis orientation must be position-dependent across the material, and formation of periodic patterns at its surface is simultaneously required. The primary challenge consists of finding the design rules to manufacture a single-piece material that presents variable physical properties without resorting to an accumulation of layers and a juxtaposition of patterns. Second, tessellated cuticles are associated with the concept of multiscale structures. Biomimetic materials must thus include anisotropic architectures at the nanoscale, micrometre scale and macroscale. Third, the double versatility of colors and patterns must ideally be achieved through a simple and single stage to make the self-organization process closer to morphogenesis. Finally, an optimal use of resources has to be targeted, as in nature: reducing the number of materials to be implemented since living organisms use only a few chemical elements (C, H, O, N, P and S, typically) to self-organize into a multitude of complex structures; avoiding metallic nanoparticles and metallic layers to realize an environmentally responsible approach; and considering the recyclability of materials from the perspective of mass-market production.

In this paper, we approach the problem of the high-fidelity capture of the structural complexity observed in nature by focusing on patterned, iridescent carapaces of insects with a complex twisted organization of chitin fibres. At a small scale (the micrometre or submicrometre scale), the helical pitch and orientation of the helical structure depend on the position in three dimensions, and the formation of macroscopic periodic patterns is required. We used LC oligomers to mimic biological LCs. We attempted to reproduce the textural, structural and color properties of these biological LCs at several length scales. We made optimal use of resources and stages during the fabrication procedure, in the spirit of eco-design. By means of a single sequence based on self-organization, precise control of a single-piece sample structure composed of two different colored patterns with the same unique pitch gradient was enabled. The functionalities of both materials are relevant to optical communication and camouflage. In addition to this set of conceptual advances, we present a concrete use of the material for advanced optical tags in cryptography.

## Results

### Lessons from nature

With the previous objectives in mind, we focus on the emblematic case of the scarab beetle *Chrysina gloriosa* (Fig. 1c), which is found from southwestern North America to Central America in pine, pine-oak, and juniper forests. The cuticle of *C. gloriosa* exhibits green and silver bands with selective and broad light reflections, respectively. The green bands exhibit a cellular polygonal texture in optical microscopy images, whereas the silver bands exhibit a non-patterned planar texture (Fig. 1d). Previous studies on the cuticle of *C. gloriosa* have reported the following: the cellular structure of the concentric rings in the green bands, as established through TEM investigations^20^ or fluorescence confocal microscopy^21^; the variation in the optical properties with the incidence angle, as established through Mueller matrix spectroscopic ellipsometry^22,23^; the existence of an array of multiwavelength micromirrors in the green bands^24^; and the generation of self-healing Bessel beams from polygonal cells^25^.

In the following, we set out the lessons learned from nature about the structure and optical response of both bands.

Cross-sectional transmission electron microscopy (TEM) views of the green and silver bands show the typical cholesteric fingerprint (stripe) texture (Fig. 1e). The axis of the helix lies in the plane of the image. The fingerprint texture of the (unlayered) cholesteric structure appears as a network of alternating bright and dark stripes. This periodic network thus arises from periodic modulation, along the helix axis, of the orientation of the molecular director (the orientation of the elongated chitin fibres depicted in Fig. 1a) and the refractive index. The helical axis is perpendicular to the stripes. The distance between two identical stripes is related to the half pitch of the twisted organization of the chitin fibres. For the green bands, the cuticle displays curved stripes in the upper part of the exocuticle, which means that the orientation of the helical axis is spatially changing, and parallel stripes in most of the endocuticle region. For the silver bands, the stripes are parallel in the whole chitinous part of the cuticle, the orientation of the helical axis is fixed. For both bands, a pitch gradient occurs from the top of the exocuticle to the bottom of the endocuticle (Supplementary Fig. 1).

Before proceeding to the analysis of the optical spectra, a preliminary comment must be made here regarding the fact that between 0 and 50% of the unpolarized incident light is reflected or transmitted, which is true for all of the reflection and transmission spectra presented in this study. Unlike common mirrors, which reverse the polarization of the light they reflect, the cholesteric planar texture reflects circularly polarized light with the same handedness as that of the twist of the helix, while light with the other polarization is transmitted^26^. This behavior is referred to as the polarization-selectivity rule, which is valid only at normal incidence. Consequently, up to 50% of the ambient unpolarized light at the selected wavelength can be reflected. The other component (50% of the ambient unpolarized light at the selected wavelength) is transmitted. Only first-order Bragg reflection is possible at normal incidence. All orders of reflection may occur at oblique incidence or when the helical structure is tilted, and the reflected or transmitted light is elliptically polarized.

The reflectance spectrum ascribed to the silver band in Fig. 1f exhibits a broadband reflection from 450 to 800 nm with a variable reflectance. This broad band can be identified from the pitch gradient shown in the TEM micrograph of a cross section (Supplementary Fig. 1b) and the related measurements, which give a pitch p varying from ~280 to 900 nm (Supplementary Fig. 2a). By taking *n* = 1.63 for the value of the average refractive index of the chitinous part of the cuticle of *C. gloriosa* (in ref. ^14^ (p. 214), the ordinary and extraordinary indices are set to 1.59 and 1.68 for *Plusiotis gloriosa* (a synonym now invalid for *C. gloriosa*), which leads to a mean refraction index equal to ~1.63.) and using the Bragg law at normal incidence, *λ*_0_ = *np*, as given in the introduction, we obtain a wavelength band of ~455–1470 nm (the measurements in Fig. 1f stop at 850 nm because of the technical limitations of the spectrophotometer). The silver band thus plays the role of a flat metallic reflector operating over the visible spectrum and into the infrared spectrum. The oscillations, or ripples, modulating the reflection spectrum in Fig. 1f can be attributed to longitudinal interferences associated with the existence of a singular region named the *emergence line* between the exo- and endo-cuticles (i.e., a trough in the periodicity variation), as observed by SEM and discussed in ref. ^24^. The presence of a ripple structure superimposed on a broadband plateau is also present in the spectrum of the gold cuticle of the scarab beetle *Chrysina aurigans*^27,28^.

The reflectance spectrum ascribed to the green band exhibits a broadband reflection from 450 to 800 nm. The pitch gradients in the silver and green bands occur in ranges of comparable amplitudes, as shown in Supplementary Fig. 2a. However, for the green band, the reflectance is very low (between 8 and 10%) from 450 to 800 nm, with a characteristic green peak at 520 nm, because the green bands act as green diffusers. Diffuse reflection occurs when light is reflected at many different angles rather than at just one angle as in the case of specular reflection. The green bands are green diffusers because the arrangement on a curved surface of polygonal cells, with curved and concave stripes (Fig. 1e and Supplementary Fig. 1a), with a spatially varying helical axis, significantly broadens the angular distribution of reflection. Both kinds of curvature (the surface of the cuticle, and the orientation of the helical axis inside each cell) remove the angular dependence of the reflection wavelength normally associated with Bragg reflection when the cholesteric material is a flat film with a pattern-free planar texture. The result is a nearly angle-independent greenish appearance of the beetle at long viewing distances, which may enable the avoidance of detection by predators in green foliage. The disruptive green-silver patterning is a good match for the habitat of *C. gloriosa* since the foliage of the juniper tree is green with white resin flecks. This situation might provide the insect with efficient camouflage^29^.

It should be noted that the reflective and absorptive properties of the wax layer at short wavelengths may influence the shape of the reflection spectrum at the beginning of the visible region, as discussed in the case of *Chrysina aurigans*^30^.

**Fig. 2:**
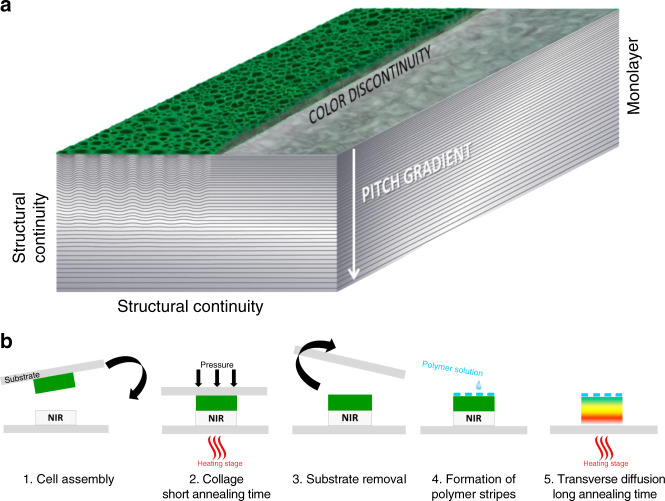
Fundamental principles to mimic the tessellated cuticle. **a** Tridimensional two-band structure to be considered. **b** Physical design in five stages.

To summarize, Fig. 2a shows a 3D view that displays the color and structure properties to be simultaneously addressed in the goal of biomimetic design. The biomimetic material must be a monolayer; exhibit a disruptive character of green and silver colors; display the continuity of the twisted structures in all directions (no disruption in the pitch distribution and in the orientation of the helical axis from region to region) with, in the upper part of the film, an alternate mix of variable and fixed orientations of the helical axis; include a pitch gradient related to reflection colors ranging from green to near IR; and exhibit pitch variations in similar ranges.

### Biomimetic design

Recent successes in utilizing soft materials for the design of advanced photonic structures promise compelling opportunities, but they rarely capture the range of structural complexity observed in natural optical materials^31^. Using synthetic LC materials for biomimetic purposes is an underutilized solution in the toolbox of researchers and engineers, partly due to challenges in fabrication, integration, and structural control at the nanoscale and microscale of soft components. However, LCs are first-rate candidates because they offer anisotropic self-organization, multiscale architectures, and a diversity of materials, such as fluids, polymers or gels, that can be inserted between two solid or flexible substrates or handled as free-standing films.

The design procedure we propose uses vitrifiable cholesteric oligomers with a siloxane backbone. The rapid cooling of samples below the glass-transition temperature, from the viscous cholesteric phase to room temperature, prevents crystallization and preserves the cholesteric structure and colors. Polysiloxanes form a group of materials with unusual properties, which may include high thermal stability, low surface energy and a low glass-transition temperature^32^. The present biomimetic design takes advantage of these properties. LC polysiloxane oligomers from Wacker Chemie GmbH were used^33^ (Supplementary Fig. 3). The molecule is a siloxane cyclic chain with two types of side chains attached via spacers: an achiral mesogen and a (chiral) cholesterol-bearing mesogen^34^. When used to coat a glass or plastic substrate, this compound exhibits typical iridescent colors that range from blue to red with the tuning of the molar percentage of chiral mesogens in the oligomer molecule, which can vary from 31 to 50%. LC polysiloxane oligomers have been the subject of various studies, including basic investigations of their reflective properties^35^, the fabrication of optical notch filters and variable bandwidth reflectors^36,37^, low-frequency mechanical relaxation measurements of molecular motions near the glass-transition temperature^38^, the color gamut of planar films and flakes based on colorimetry^39^, the electro-optical behavior of cholesteric flakes^40^, mechano-optical effects^41^, lasing from dye-doped materials^42^, the self-organization of metallic nanoparticles into various cholesteric textures^43,44^ and the control of the stretching and compression of ultrashort laser pulses^45^.

Polygonal textures, consisting of arrays of micrometre-scale polygonal cells (Supplementary Fig. 4a), can be observed in flat CLC films when the helical axes are strongly tilted with respect to the substrates. This situation can be observed in an open film via hybrid anchoring of molecules, which preferentially align tangentially to the solid substrate on which the CLC is coated (planar anchoring conditions) and perpendicularly to the air interface (perpendicular anchoring conditions). Supplementary Fig. 4b shows a three-dimensional representation of the polygonal texture obtained by combining various microscopy methods and provides more information on the formation of the polygonal texture in the materials under study. Polygonal fingerprint textures were first imaged via optical microscopy in large-pitch low-molar-mass CLCs in the early 1970s^46,47^. In the mid-1990s, polygonal textures in the film plane and in cross sections were observed via TEM^48^ and AFM^49,50^. In the 2010s, investigations of polygonal textures focused on glass-forming oligomers^51–57^ such as those used in the present study, which does not focus on the polygonal texture itself.

The biomimetic design is based on the control of the thermally driven diffusion between one green and one infrared light-reflecting layer accompanied by local changes in the molecular anchoring conditions in specific regions of the film. It involves five stages (Fig. 2b). Stage 1: A cell made of cholesteric films coated on glass substrates facing each other, reflecting the light in the green and IR spectrum, respectively, is assembled at RT. Stage 2: The sandwich cell is heated at 120 °C for 30 s to adhere the films together. Stage 3: After quenching of the sample at RT, the upper substrate is removed. Stage 4: Spaced bands of an isotropic polymer, polyvinyl alcohol (PVA), in solution in water are deposited on the free surface of the bilayer film. Stage 5: After drying of the aqueous solution, the bilayer film is heated in the cholesteric phase at 120 °C for 1 h. Three major events simultaneously occur due to the thermally driven diffusion between the two films in the direction perpendicular to their surfaces: a physical transverse pitch gradient is built as the result of the chemical gradient in the chiral species; the polygonal texture nucleates in the regions in contact with air between the polymer bands (the anchoring of molecules becomes perpendicular in the upper part of these regions), whereas the planar texture is preserved in the regions below the polymer bands (the anchoring of molecules is kept as planar in these regions); and the bilayer system becomes a single layer. Finally, the film is quenched at RT. The structural and optical properties are maintained in the solid glassy film.

The fifth stage of the design is the crucial, one-step, stage. By offering a single sequence based on self-organization, precise control of the sample structure composed of two different colored bands with the same unique pitch gradient is enabled. An association of the alternate green and silver bands was obtained by drawing spaced bands on the free surface of the film before the thermal diffusion began using a pencil dipped in PVA solution. Alternatively, band-shaped adhesive tape could be fixed on the surface of the film, which is then spin-coated with the solution (see Methods). The tape was removed before the solution was completely dried. Many patterns other than bands can thus be drawn, which offers a wide, choice on demand for designing reflective patterns. PVA is commonly used in the field of thermotropic LCs to induce a molecular orientation parallel to the support^58^. Here, it was used to protect specific regions targeted to become silver bands from air. By analogy, it protects the film from texture evolution (planar to polygonal texture transition), while the wax layer visible in Fig. 1e and Supplementary Fig. 1 protects the chitinous part of the cuticle from the external environment, with the difference being that the polymer layer only covers the silver bands, while the wax layer covers the green and silver bands. Finally, the PVA bands can be dissolved in water if required. Patterning techniques, such as metallic layer sputtering deposition^31^, moulding^59^, alignment methods^60^ or lithography^61,62^, would require many stages to achieve the present patterning objectives and would lead to discontinuous layers and structures. In addition, the range of producible patterns, the properties of surface coatings related to these techniques and their durability are supposedly restricted^6^.

### Biomimetic samples: texture, structure and optical behavior

**Fig. 3:**
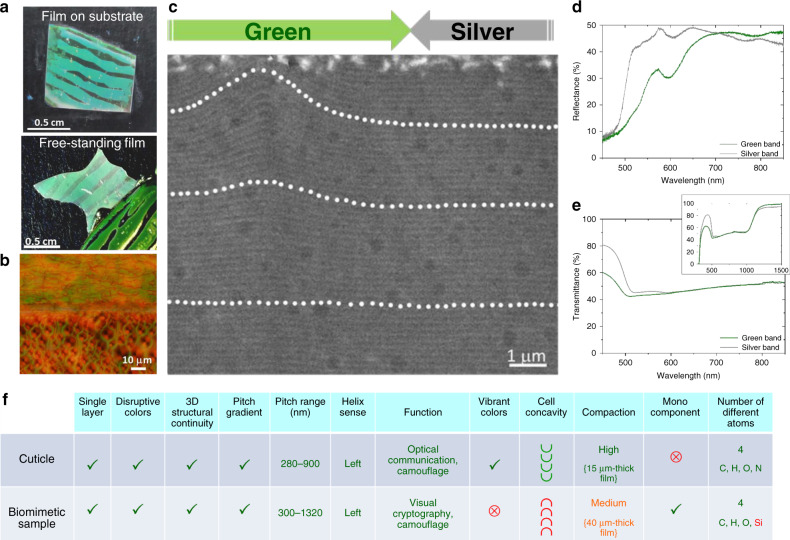
Features of the biomimetic samples. **a** Biomimetic samples on a glass substrate and as a free-standing film. **b** Optical micrograph of the interfacial region between silver (top) and green (bottom) bands (reflection mode, unpolarized light). **c** TEM image of transverse structures at the interface between green and silver bands and close to the top surface. White dots underline the stripes of the fingerprint texture. The transition between curved and straight stripes is observed to be very gentle. **d** Reflectance spectrum of green and silver bands. **e** Transmittance spectrum of green and silver bands with the same wavelength scale as in **d**. Inset: the same spectrum displayed with the full operating wavelength band. **f** Multicriterion comparative table of features of the cuticle and biomimetic sample. The mentions in green (resp. red) report a similarity (resp. discrepancy). The mention in orange corresponds to an in-between situation.

Figure 3a shows the biomimetic samples when coated on a glass substrate or as a free-standing film. The silver bands appear as black because the film (coated on a glass substrate) is placed on a black substrate and transmits light. It is semitransparent: indeed, a cholesteric film reflects at most 50% of unpolarized ambient light (as explained previously). The perception of colors in the macroscopic images is highly dependent on the ambient lighting conditions and on the viewing angle. This is the reason why, in Fig. 1c, some silver bands of the beetle partially appear as black: the cuticle is curved, and the light reflected by the specular mirrors illuminated by the flash on part of the curved surface is not collected by the camera. On the other hand, all of the green bands are seen as green because the light reflected by the green bands is diffusely reflected. This is a consequence of the existence of an array of polygonal cells in which the orientation of the helical axis is spatially variable. Magnified views of a set of green and silver bands for which the whitish aspect of the silver band is more visible are given in Supplementary Fig. 5. Additional comments regarding the differences in the perceived colors, from the biological to the biomimetic material, will be given below in the discussion of the optical spectra.

Biomimetic samples consist of a single piece that does not include any foreign material. By analogy, solidification by thermal quenching of the viscous material, at the end of the ultimate fifth stage, plays the role (transition from a soft to an hard material) of cuticle sclerotization^63^ after the cuticle has expanded to its final size and shape.

The optical micrograph of the cuticle (Fig. 3b) shows a well-defined boundary between bands, which is more pronounced than in the biological material (Fig. 1d). At the sub-micrometre scale, TEM investigations of cross sections (Fig. 3c) show that the transition between bands is very gentle in the case of the biomimetic material. This set of observations validates the continuity of the transverse structures and the role of the orientation of the helical axis—from tilted to perpendicular—in the macroscopic color differences. The texture periodicity—related to the helical half-pitch—as a function of the film depth is determined from cross-sectional TEM images (Supplementary Fig. 6). The pitch ranges in the biological—ca. 280–900 nm (Supplementary Fig. 2a)—and biomimetic—ca. 300–1320 nm (Supplementary Fig. 2b)—materials are comparable in their orders of magnitude.

The reflection spectra ascribed to the biomimetic silver and green bands (Fig. 3d) have comparable shapes. Both bands behave as broadband reflectors from ~500 to 850 nm. This broad wavelength range is comparable to that of the cuticular silver band in Fig. 1f. Some differences in reflectance between the biomimetic silver and green bands are apparent: the spectrum ascribed to the silver band exhibits enhanced reflectance from 500 to 700 nm because here, the green band continues to exhibit diffuse reflection, whereas the silver band behaves as a specular mirror. However, the reflectance of the biomimetic green band is much more important than that of the cuticular green band. A green peak at 570 nm is observed for the biomimetic green band, whereas the corresponding peak is located at 520 nm for the cuticular band. This distinction contributes to the difference in the perceived green reflection color between the biological material and the biomimetic one. Other reasons, of a conceptual nature, may also be invoked to explain why the reflection colors and optical textures of the biomimetic samples are much less vibrant than those of C*. gloriosa*. First, the chemical compositions of the biomimetic and biological materials are very different. In some beetles, the presence of uric acid in the chitin matrix plays a major role in imparting color by enhancing the birefringence and reflectivity^7,14^. Second, the thin and transparent wax layer may play a role in the perception of colors. It may scatter light and contribute to the shiny appearance of some beetles^29^. In the case of the cuticle of *Chrysina boucardi*, the overall chromatic effect has been described as deeper in color because the cuticle is covered with wax^15^, a situation that was noted to be evocative of the metal flake paint used on automobiles.

While the opacity of the cuticle prevents the measurement of light transmission, the transmission spectra of the biomimetic sample show a broad bandgap from 500 to 1000 nm (inset in Fig. 3e). The fact that the plateau begins at 500 nm is consistent with the sudden increase in reflectance beginning at ~500 nm in Fig. 3d. The transmittance spectra for both bands are very similar, providing further confirmation that the difference in reflection colors is due to the dual presence of variable and fixed orientations of the helical axis and not to significant pitch variations in the bulk of the material. This broad band is objectified by the pitch gradient shown in the TEM micrograph of a cross section and the related measurements, which give a pitch varying from 300 to 1320 nm (Supplementary Fig. 2b). By taking *n* = 1.5 for the value of the average refractive index of the polysiloxane film^64^ and by using the Bragg law *λ*_0_ = *np*, at normal incidence, we obtain a wavelength band of ~450–1980 nm. The wavelengths at the left edge of the Bragg band are comparable: 500 nm, as displayed in Fig. 3e, vs. 450 nm, as calculated. A wavelength discrepancy appears at the right edge: 1000 nm vs. 1980 nm. We assume that the number of helix turns ascribed to the twist from 666 nm (1000 nm:1.5) to 1320 nm (1980 nm:1.5) is too low to allow a contribution to the Bragg band from this part of the pitch gradient. The reflectance indeed depends on the number of pitches (i.e., the number of helix turns), which means that the twist needs to develop within a minimal film thickness to make possible the appearance of a Bragg peak that is detectable under standard spectrophotometry conditions^65^. Our hypothesis is supported by the sudden jump in the half-pitch in the 333–660 nm range for a small sample thickness of ~6 μm (vs. the total sample thickness of 40 μm), as shown in Supplementary Fig. 2b. This thickness cannot permit a sufficiently large number of helix turns and the detection of related Bragg reflections.

### Multicriterion comparison

A criterion-by-criterion comparative table of the cuticle and biomimetic sample is given in Fig. 3f. Eight of twelve criteria are rated as high. The four criteria learned from the physical study of the biological material and reported in Fig. 2a as essential to claim the biomimicry status of the sample include the monolayer status, 3D structural continuity, 2D color discontinuity, and transverse pitch gradient. The biomimetic sample is chemically a monocomponent, while the cuticle is a chitin protein composite. The compaction can be taken as high in the cuticle and as medium in the biomimetic sample because the combination of common physical properties occurs within thicknesses equal to ca. 15 and 40 μm, respectively. Two characteristics are clearly different: reflection colors are vibrant in the cuticle and much less vibrant in the biomimetic sample, and polygonal cells are concave in the cuticle and convex in the biomimetic sample.

### Biomimetic applications in optical communication

The twisted CLC patterns issued from the present design pave the way to many possibilities of practical uses.

They may inspire future applications in optics, in the field of non-specular properties such as deflection and lensing in geometric phase planar optics^66–68^. The phase of light that is Bragg-reflected off the helical structure can be controlled over 0–2π depending on the spatial phase of the helical structure^66^. The manufacture of planar elements with arbitrary reflected wave fronts via orientation control is thus desired. This requires the helix phase to vary depending on position, which objective is reached with on-demand chiral patterns provided by the present design.

Insects and humans have common concerns in terms of energy regulation, optical communication and camouflage^69^. Concerning the last two functions, the fabrication of biomimetic samples inspires us to design tags in the field of cryptography and encryption^70,71^. Graphically and spectrally encoded tags have become appealing for carrying information about tagged products because of their high coding capacity and wide applicability^71^. Incorporating anti-counterfeiting tags with physical unclonable functions (PUFs) into objects is a promising solution for their authentication. Ongoing research hints at future LC-based technologies that may benefit from the peculiar behavior of CLCs subject to curvature, as reported for the case of cholesteric shells and droplets with the objective of generating unclonable patterns^72–74^.

**Fig. 4:**
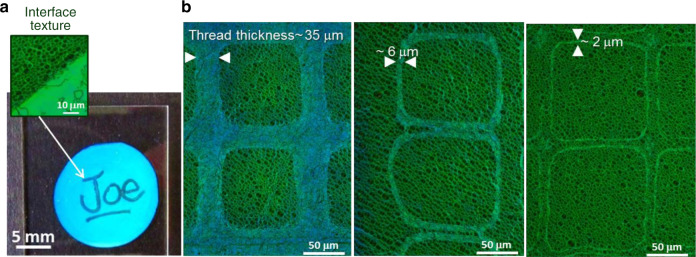
Practical uses in visual cryptography. Multiscale optical tags with taggants from the **a** millimetre to **b** micrometre scale.

We provide two physical methods in relation to the size of the pattern to code and the technical solution to be implemented: the objective consists of encoding and hiding a signature (taggant) with different colors at different scales, from the millimetre to micrometre scale (Fig. 4), with an optical readout visible to the naked eye (Fig. 4a) or by optical microscopy (Fig. 4b).

For millimetre-size taggants (Fig. 4a), the free surface of the green film is signed by the user with a pencil dipped into a polymer (PVA) solution. After water evaporation, the signature is invisible to the naked eye (Supplementary Fig. 8) and difficult to discern with the help of a microscope since the polymer film is thin, isotropic and transparent. It is revealed after thermal treatment (120 °C for 1 h) as the consequence of the nucleation and growth of the polygonal texture in the polymer-free parts of the film. The difference between the planar (“Joe” signature) and polygonal textures leads to color differences. Micrometre-size taggants (Fig. 4b) can be formed by fixing a copper grid to the film and depositing a polymer solution on the grid–film system. The grid is then removed, and the film is annealed. Grid patterns of taggants with geometrical features at different scales, from several tens of micrometres to a few micrometres, are revealed after thermal treatment and can be achieved by using different spin-coating conditions to deposit the polymer solution (see Methods), or grids with different geometrical features. Glass-forming CLCs are easily malleable, and their structure can be locked in solid paint after quenching. Solidified hybrid textures can be formed on a support that can be safely applied on an object by printing it on a plastic film or embedding it in a resin. Under illumination, the tags reveal patterns that, according to the taggant size, are visible to the naked eye, without or with the use of polarizers, or detectable by optical microscopy. The hybrid planar-polygonal texture provides the possibility of forming an image (the PUF tag) when it is embedded in a polygon-textured matrix, producing a specific optical response (the PUF pattern), which has to be recorded in a list of PUF keys (a database). An assembly of cells carries an additional level of PUF since the relative positions, sizes and optical patterns of cells are unique, not clonable and unpredictable. *C. gloriosa* may display information and hide in the background. Therefore, the comparable role of artificial tags and biological patterns brings an additional criterion to the biomimetic nature of the present biomimetic samples in terms of physical functions.

By using CLC oligomers we have engineered a single-layer material mimicking the striped cuticle of the scarab beetle *C. gloriosa*. Textural, structural and color properties were reproduced at several length scales. The functionalities of both materials were relevant to the topics of optical communication and camouflage. A multicriterion comparison revealed a very high level of biomimicry. The double versatility of colors and patterns was achieved during a simple and single stage based on a thermally driven self-organization process. The fabrication of biomimetic samples has inspired us to design proof-of-concept prototypes of optical tags in the field of cryptography and encryption. The design strategy involves an optimal use of resources in the spirit of eco-design, and a high versatility of chiral patterns unreached by the current manufacturing techniques.

## Methods

### Biological samples

A male specimen was collected at Madera Canyon (Santa Cruz County, Arizona, USA) in July 2014. The cuticle samples including green and silver bands correspond to the elytra and were removed with a razor blade.

### Synthetic materials

LC vitrifiable side-chain oligomers with a siloxane backbone were obtained from Wacker-Chemie GmbH, Munich, Germany (Supplementary Fig. 3).

### Alignment and sacrificial layers used in the manufacture of cholesteric films

A PVA layer formed on the glass substrates supporting the cholesteric films was used as a promoter of the Grandjean planar texture or as a sacrificial layer to obtain free-standing films after the sample was placed in water. PVA as a powder (P8136, Sigma-Aldrich) was dissolved at 4 wt.% in distilled water. The aqueous solution was deposited on the substrate using a spin coater (Ossila Ltd.). The thickness of the dried film was ~2 μm. A single sequence of spin-coating consisted of the deposition of 1.30 ml of solution during a program consisting of three sequences with the following speed rates and durations: 5000 rpm@20 s—4000 rpm@40 s—3000 rpm@60 s. This operation was repeated three times.

### Preparation of individual films for the biomimetic samples

Two distinct green-reflecting (SG) and infrared-reflecting (IR) samples were prepared on a heating stage at 120 °C. SG was used as provided by the supplier. IR was a blend made with 40 wt.% of a red-reflecting compound (SR) and 60 wt.% of a nematic compound (SN). A 50-μm-thick film of SG was coated with a razor blade on a 150-μm-thick cover plate. The sample was then quenched at room temperature (RT). The mean reflection wavelength of the film was 520 nm (Supplementary Fig. 7). A 20-μm-thick IR film was sandwiched between a 1-mm-thick glass plate and a 150-μm-thick cover plate separated by 20-μm-thick spacers. The sample was then quenched at RT. The mean reflection wavelength of the film was 1600 nm (Supplementary Fig. 7). The cover plate was sharply removed from the film with the aid of pressurized air to avoid the formation of scratches at the surface of the film. That the free surface of the film remained smooth after the operation was checked by optical microscopy. When the realization of free-standing samples was the goal, the glass plate that supported the IR film was previously covered with an 80-μm-thick PVA film made from a 30 wt.% solution. The sample was then placed in a vial of distilled water. It detached from the plate in <1 h at RT. The PVA bands on the top surface also dissolved in water. The sample thus consisted of a monolithic piece of glassy polymer that did not include any foreign material.

### Formation of bands in the biomimetic samples

To draw bands, an alternate solution to the use of a pencil dipped in PVA solution was to place 75-μm-thick dicing tape (6033, Loadpoint), cut with dimensions close to those of the green bands of *C. gloriosa*, on the free surface of the bilayer film (Supplementary Fig. 9), at the end of stage n°3 in Fig. 2b. The surface of the sample with tape (typical size: 1.5 cm^2^) was covered with 1.30 ml of PVA solution and spin-coated according to a program consisting of three sequences with the following speed rates and durations: 5000 rpm@20 s—4000 rpm@40 s—3000 rpm@60 s. This operation was repeated three times. The tape was gently removed from the film with the help of tweezers before the PVA film was completely dry.

### Preparation of functional films for optical cryptography at the micrometre scale

A copper grid (1GC 200 Pelco, Ted Pella) with square patterns (hole width = 90 μm, bar width = 37 μm) was fixed with adhesive tape on a 20-μm-thick SG open film. A quantity of 1.30 ml of PVA solution was spin-coated on the free surface of the film at 900 rpm for 6 s for a variable number of sequences, therefore obtaining a variable quantity of solution. The promotion of one type of pattern size at the detriment of other size depended on this process. The thicknesses of the thread patterns (related to the grid bars—the solution was trapped by capillarity below them) with mean sizes of 35, 6 and 2 μm (Fig. 4b) corresponded to 6, 4 and 3 sequences. The grid was then removed, and the sample was annealed at 120 °C for 1 h.

### Inclusions of samples for ultramicrotomy

A small piece of material was embedded in EMBed–812 resin (Electron Microscopy Sciences), which was then cured at 62 °C for 2 days (biological sample) or at 37 °C for 11 days (biomimetic sample).

### Preparation of TEM samples by ultramicrotomy

A diamond knife at ambient temperature was used to cut 80-nm-thick ultrathin slices with a Leica UCT ultramicrotome. The material was cut perpendicular to the film surface (cross sections). Slices were placed on single-slot Formvar-coated copper grids (GS2x1-C3, Gilder Grids Ltd.).

### TEM conditions

A Jeol JEM–1400 microscope operating at 80 kV equipped with a Gatan Orius SC1000B CCD camera was used.

### Periodicity profiles from TEM images

Quantitative image analysis was performed with DigitalMicrograph software from Gatan. The intensities of the gray levels on a given surface of the images were analysed using the Profile option.

### Optical micrographs

Textures were photographed with an Olympus BX51 stereomicroscope equipped with an Olympus DP73 digital camera.

### Optical measurements

The reflectance and transmittance spectra of materials were collected at RT and normal incidence with unpolarized incident light. An HR2000CG-UV–NIR spectrophotometer from Ocean Optics (reflectance spectra) or a Cary 5000 UV–Vis–NIR spectrometer from Varian (transmittance spectra) was used. Baselines were recorded with air (resp. a mirror) in the beam path for transmittance (resp. reflectance) measurements.

### Reporting summary

Further information on research design is available in the Nature Research Reporting Summary linked to this article.

## Supplementary information

Supplementary Information

Reporting Summary

## Data Availability

The data supporting the findings of this study are available within the paper and its Supplementary Information file and from the corresponding author upon reasonable request.
