# Supplementary material for: Biomimetic design of iridescent insect cuticles with tailored, self-organized cholesteric patterns
Source: Nat Commun. 2020 Aug 14;11:4108. doi: 10.1038/s41467-020-17884-0 (PMC7429863; doi:10.1038/s41467-020-17884-0)
Supplement: Supplementary file 1 — Supplementary Information [file 41467_2020_17884_MOESM1_ESM.pdf]

Supplementary Information

Biomimetic design of Iridescent Insect Cuticles with Tailored, Self-Organized Cholesteric Patterns

Adriana Scarangella, Vanessa Soldan and Michel Mitov

Contents

1. TEM transverse images of the cuticle ascribed to the green and silver bands.
2. Half-pitch as a function of depth for the green and silver bands of the cuticle and biomimetic sample.
3. Liquid crystalline oligomers: chemical formula and main features.
4. On the polygonal cholesteric texture.
5. Magnified views of biomimetic samples under different conditions of illumination.
6. TEM transverse image of the biomimetic sample ascribed to the green and silver bands.
7. Transmission spectra of individual green-reflecting (SG) and infrared-reflecting (IR) films used for the manufacture of biomimetic samples.
8. Disclosure of the "Joe" taggant signed by the user with a pencil dipped into a polymer (PVA) solution.
9. Biomimetic sample with spaced dicing tapes fixed on its free surface, just before the deposit of the PVA solution.

References

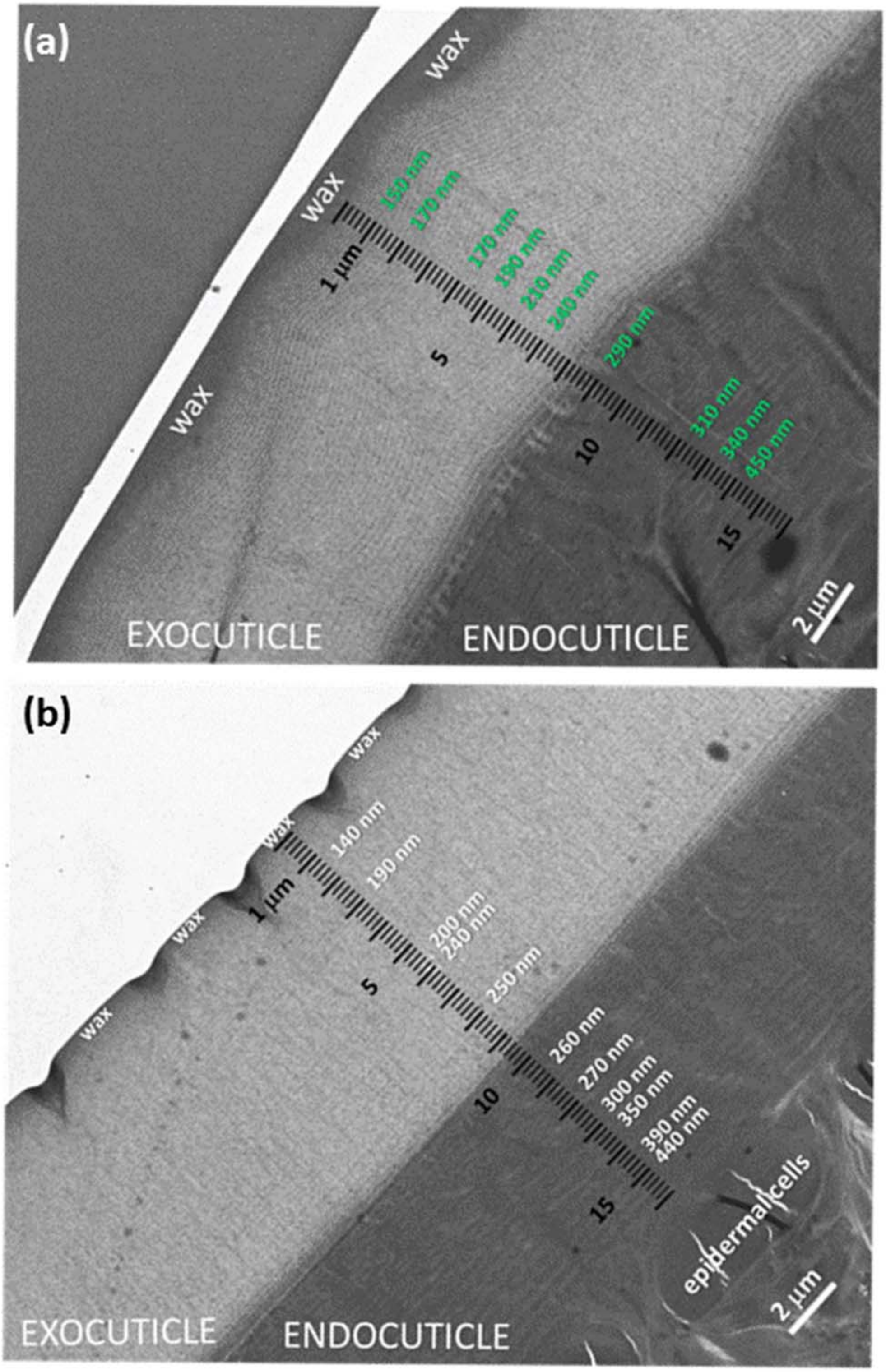

Supplementary Fig. 1. TEM transverse images of the cuticle ascribed to the **a,** green and **b,** silver bands. For the green band, the measurements are made vertical to the apex of a

polygonal cell. For both bands, measurements are stopped in the endocuticle when the TEM contrast between dark and bright stripes in the fingerprint texture becomes too low to allow reliable measurements. The cholesteric organization propagates over a short length (<2 μm) beyond the last value (14.3 μm) that we chose to display for the texture periodicities in Supplementary Fig. 2a and up to the narrow deposition zone (also named the subcuticle) between the end of the endocuticle and the epidermal cells [1].

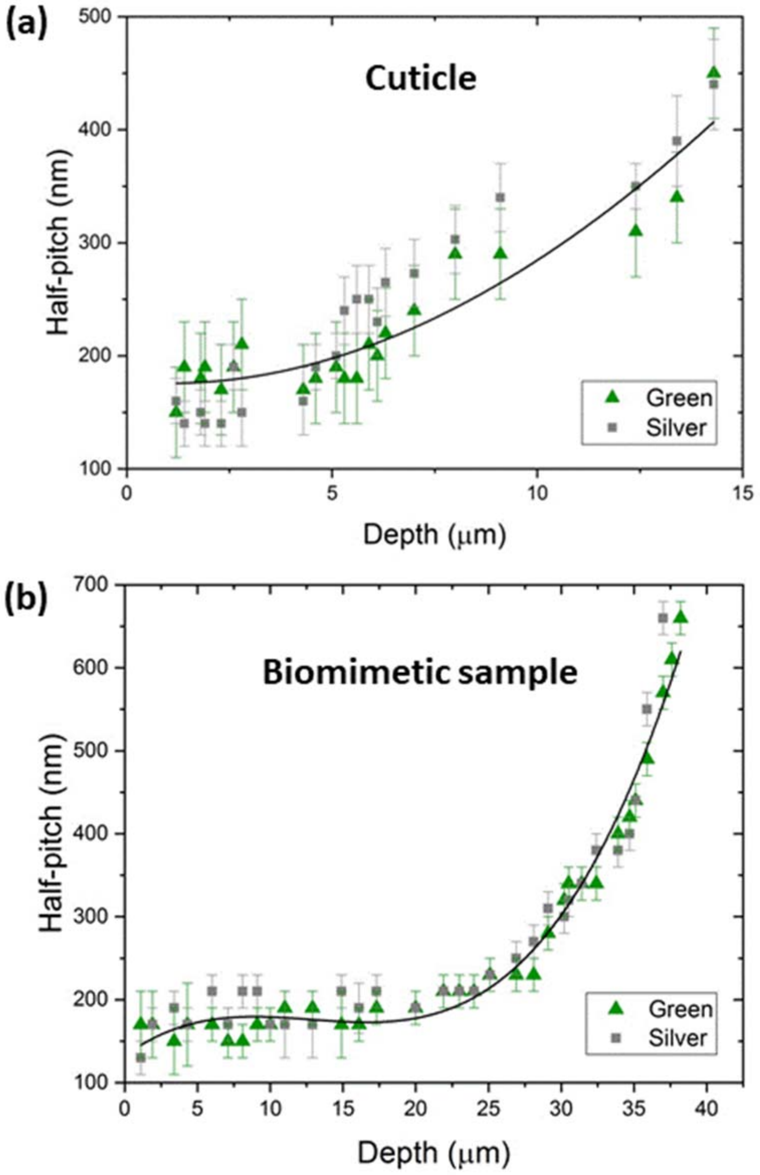

Supplementary Fig. 2. Half-pitch as a function of depth for the green and silver bands of the a, cuticle and b, biomimetic sample, as determined from image analysis of the TEM micrographs shown in Supplementary Fig. 1 and Supplementary Fig. 6, respectively. Error bars correspond to the dispersion range.

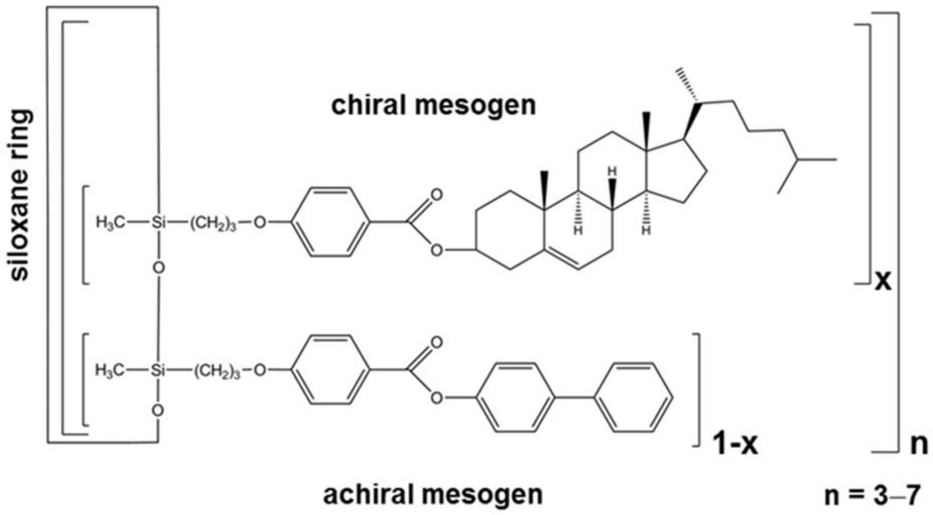

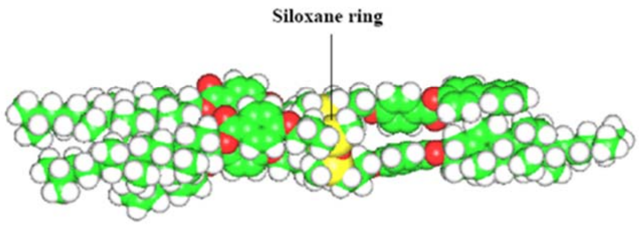

Tetrasiloxane with 1 biphenyl unit and 3 cholesterol units

	Blue	Green	Red
x = molar percentage in chiral mesogens	50	44	31
Bandgap λ_{min}–λ_{max} (nm)	420–470	480–560	670–750
Glass transition temperature (°C)	40-50	45-55	40-50
Clearing temperature (°C)		180-210	

Supplementary Fig. 3. Liquid crystalline oligomers: chemical formula and main features. Liquid crystalline polysiloxane oligomers consist of a siloxane ring to which two types of side chains are attached via aliphatic spacers: an achiral mesogen and a chiral cholesterol-bearing mesogen [2]. The pitch depends on the molar percentage of chiral mesogens in the molecule. Via chemical engineering, different percentages therefore give rise to different films with a reflection colour ranging from blue to red. To this set of chiral oligomers is

added a nematic oligomer, silicon nematic (SN), only bearing achiral mesogens. The liquid crystal phase appears between 180–210°C (clearing temperature range) and 40–55°C (glass transition temperature range). The helical structure is left-handed.

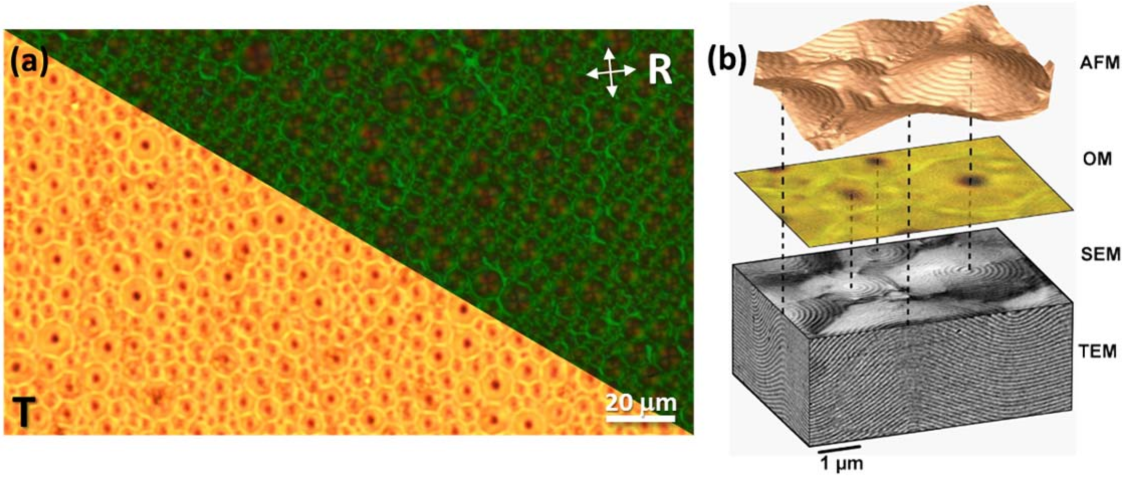

Supplementary Fig. 4. On the polygonal texture. a, Polygonal cholesteric textures observed in the synthetic materials of the present study by optical microscopy in transmission (unpolarized light) and reflection modes. The white arrows indicate the orientations of the crossed polarizers for the reflection mode. **b,** Tridimensional structure of the polygonal texture obtained by combining different microscopy methods: atomic force microscopy (AFM), optical microscopy (OM), scanning electron microscopy (SEM) and transmission electron microscopy (TEM) [3]. ©The authors 2011. The dotted lines link polygon vertices. AFM imaging reveals the cone shape of the polygons, which are the locus of the double-spiral patterns. The relief of conical polygons (their height is in the 20-100 nm range) results from the competition between the surface energy and the bulk free energy. The surface tension minimizes the surface area by rearranging the fluid interface from a planar state to a polygonal field [4]. The energy is lowered by transforming the free surface into a cone, thus reducing the bulk distortion energy at the cost of the surface energy. It should be noted that the formation of a series of valleys and crests at the free surface of the LC films with antagonist anchoring is also observable in nematic LC films [5]. The double spiral structures correspond to the adaptation of the cholesteric structure to the relief and the anchoring conditions at the limits: rod-like molecules preferentially align tangentially to the substrate

and perpendicularly to the air interface. Close to the air interface, the twisted structure cannot readily adapt to the anchoring conditions. One solution to this boundary problem is the creation of a series of alternating disclination lines at the surface [6]. Nested-arc patterns as seen in the TEM image develop because the anchoring at the free surface favors an oblique orientation of the helical axis relative to the surface. Such a situation corresponds to the director distribution of least elastic energy which allows the surface anchoring together with the bulk planar structure.

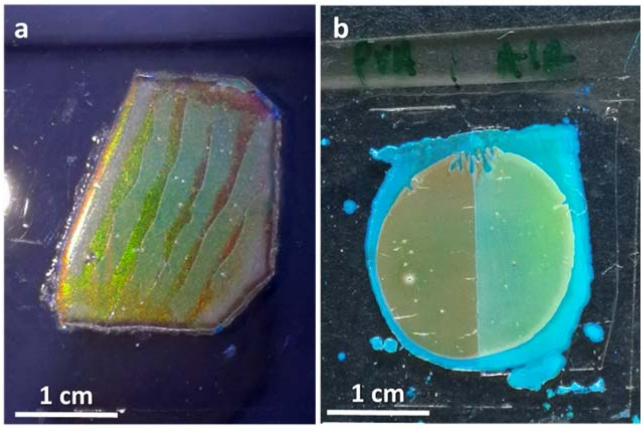

Supplementary Fig. 5. Magnified views of biomimetic samples under different illumination conditions (orientation of the light source, distance between the sample and the source, source intensity) to present the contrast between the green bands and silver (whitish) bands. Both materials are open films formed on a glass substrate. **a** Multiband sample fabricated by using dicing tapes (Supplementary Fig. 9) during the deposition of a PVA film. **b** Two-band sample.

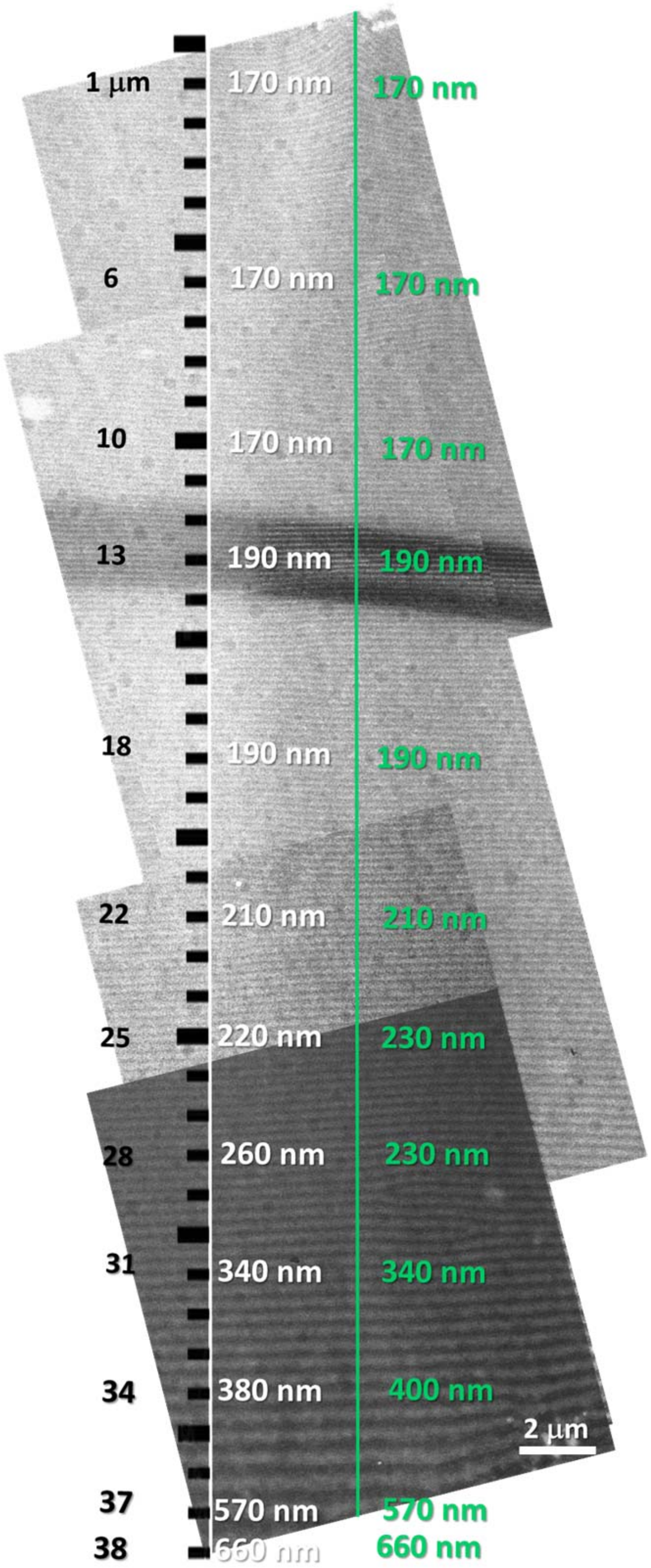
1 μm
170 nm
170 nm
6
170 nm
170 nm
10
170 nm
170 nm
13
190 nm
190 nm
18
190 nm
190 nm
22
210 nm
210 nm
25
220 nm
230 nm
28
260 nm
230 nm
31
340 nm
340 nm
34
380 nm
400 nm
2 μm
37
570 nm
570 nm
38
660 nm
660 nm

Supplementary Fig. 6. TEM transverse image of the biomimetic sample ascribed to the green and silver bands, here contiguous. For the green band, the measurements are made vertical to the apex of a polygonal cell.

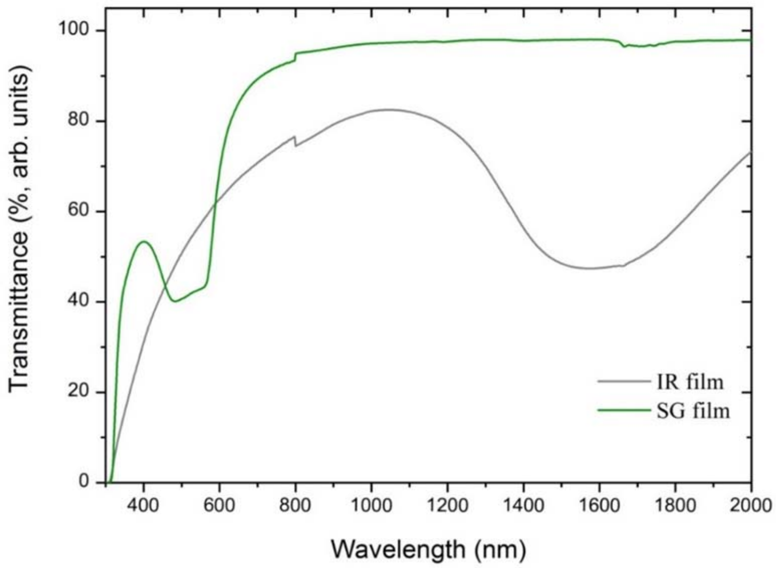

Supplementary Fig. 7. Transmission spectra of individual green-reflecting (SG) and infrared-reflecting (IR) films used for the manufacture of biomimetic samples.

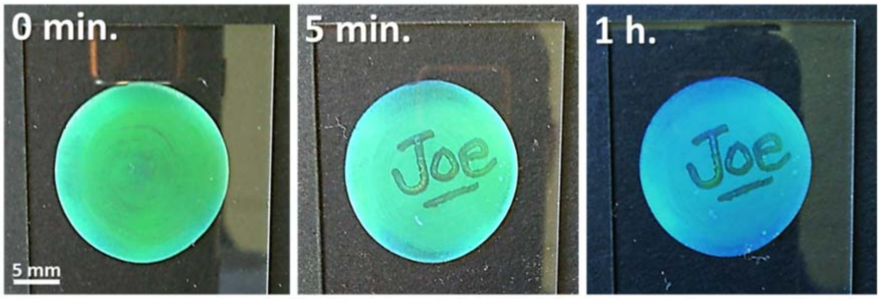

Supplementary Fig. 8. Disclosure of the “Joe” taggant signed by the user with a pencil dipped into a polymer (PVA) solution before thermal treatment and after 5 min and 1 h of thermal treatment at 120°C. The green film coated on a glass slide was placed on a black substrate and observed at room temperature and at normal incidence (the reflections on the glass slide correspond to the rectangular lens of the smartphone). The “Joe” taggant was not

visible before thermal treatment. After thermal treatment, a green region became apparent around the taggant, corresponding to the polygonal texture. Its colour then changed from green (at 5 min.) to blue (at 1 h.). This blue shift is due to the increase in the angle between the film surface normal and the orientation of the helical axis inside each cell in the polygonal texture. This phenomenon has been described in detail in Ref. [3].

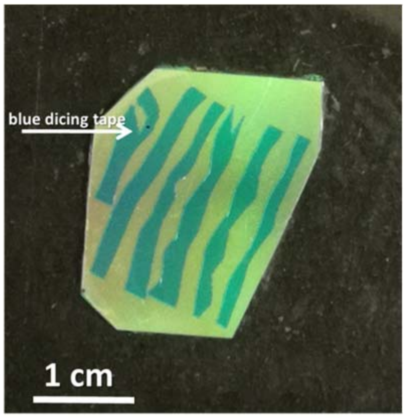

Supplementary Fig. 9. A biomimetic sample with spaced dicing tapes fixed on its free surface before the deposition of the PVA solution. To draw bands, an alternate solution to the use of a pencil dipped in PVA solution is to place (blue-coloured) dicing tapes, cut with dimensions close to those of the green bands of *C. gloriosa*, on the free surface of the bilayer film to be patterned. The regions between the tapes will be covered with the PVA solution. After drying, the thin polymer film will protect these regions, which are destined to become silver bands after annealing, from air. The dicing tapes will be removed from the sample before the fifth stage (Fig. 2b).

References

1. Andersen, S. O. Cuticle, in *Encyclopedia of Insects—2nd Edition* (eds. Resh, V. H. & Cardé, R. T.) 245-246 (Academic Press, Elsevier, 2009).
2. Kreuzer F.-H. *et al*. Cyclic Liquid Crystalline Siloxanes—Chemistry and Applications, in *Organosilicon Chemistry III* (eds. Auner, N. & and J. Weiss, J.) 566-586 (Wiley-VCH, Weinheim, 1997).

3. Agez, G., R. Bitar, R. & Mitov, M., Color selectivity lent to a cholesteric liquid crystal by monitoring interface-induced deformations. *Soft Matter* **7**, 2841-247 (2011).
4. Bouligand, Y. Recherches sur les textures des états mésomorphes — Les champs polygonaux dans les cholestériques. *J. Phys.* **33**, 715-736 (1972).
5. de Gennes, P.-G. & Prost, J. *The Physics of Liquid Crystals*, 174-176 (Oxford University Press, Oxford, 1993).
6. Saupe, A. Disclinations and Properties of the Director field in Nematic and Cholesteric Liquid Crystals. *Mol. Cryst. Liq. Cryst*. **21**, 211–238 (1973).
