# Supplementary material for: Biomimetic design of iridescent insect cuticles with tailored, self-organized cholesteric patterns
Source: Nat Commun. 2020 Aug 14;11:4108. doi: 10.1038/s41467-020-17884-0 (PMC7429863; doi:10.1038/s41467-020-17884-0)
Supplement: Supplementary file 2 — Reporting Summary [file 41467_2020_17884_MOESM2_ESM.pdf]

nature research

Corresponding author(s): Michel Mitov

Last updated by author(s): Jul 12, 2020

Reporting Summary

Nature Research wishes to improve the reproducibility of the work that we publish. This form provides structure for consistency and transparency in reporting. For further information on Nature Research policies, see our Editorial Policies and the Editorial Policy Checklist.

Statistics

For all statistical analyses, confirm that the following items are present in the figure legend, table legend, main text, or Methods section.

n/a	Confirmed	
☐	☒	The exact sample size (n) for each experimental group/condition, given as a discrete number and unit of measurement
☐	☒	A statement on whether measurements were taken from distinct samples or whether the same sample was measured repeatedly
☒	☐	The statistical test(s) used AND whether they are one- or two-sided<br>*Only common tests should be described solely by name; describe more complex techniques in the Methods section.*
☒	☐	A description of all covariates tested
☒	☐	A description of any assumptions or corrections, such as tests of normality and adjustment for multiple comparisons
☐	☒	A full description of the statistical parameters including central tendency (e.g. means) or other basic estimates (e.g. regression coefficient) AND variation (e.g. standard deviation) or associated estimates of uncertainty (e.g. confidence intervals)
☒	☐	For null hypothesis testing, the test statistic (e.g. F, t, r) with confidence intervals, effect sizes, degrees of freedom and P value noted<br>*Give P values as exact values whenever suitable.*
☒	☐	For Bayesian analysis, information on the choice of priors and Markov chain Monte Carlo settings
☒	☐	For hierarchical and complex designs, identification of the appropriate level for tests and full reporting of outcomes
☒	☐	Estimates of effect sizes (e.g. Cohen's d, Pearson's r), indicating how they were calculated

Our web collection on statistics for biologists contains articles on many of the points above.

Software and code

Policy information about availability of computer code

Data collection	OOIBase32 from Ocean Optics.<br>Cary WinUV from Agilent Technologies.
Data analysis	DigitalMicrograph software from Gatan. Version 2.32.888.0.<br>Microsoft Excel.<br>Microsoft Powerpoint.

For manuscripts utilizing custom algorithms or software that are central to the research but not yet described in published literature, software must be made available to editors and reviewers. We strongly encourage code deposition in a community repository (e.g. GitHub). See the Nature Research guidelines for submitting code & software for further information.

Data

Policy information about availability of data

All manuscripts must include a data availability statement. This statement should provide the following information, where applicable:

- Accession codes, unique identifiers, or web links for publicly available datasets
- A list of figures that have associated raw data
- A description of any restrictions on data availability

The data supporting the findings of this study are available within the paper and its Supplementary Information file and from the corresponding author upon reasonable request.

Field-specific reporting

Please select the one below that is the best fit for your research. If you are not sure, read the appropriate sections before making your selection.

- [x] Life sciences
- [ ] Behavioural & social sciences
- [ ] Ecological, evolutionary & environmental sciences

For a reference copy of the document with all sections, see nature.com/documents/nr-reporting-summary-flat.pdf

Life sciences study design

All studies must disclose on these points even when the disclosure is negative.

Sample size	One single specimen of beetle. Four fragments of its carapace were investigated. The sample size was sufficient to give evidence to the observation of its internal structure by TEM and to the optical measurements.
Data exclusions	No data exclusions.
Replication	All attempts at replication were successful.
Randomization	Randomization is not relevant to our study because the sample size is small. We used tests appropriate to small samples.
Blinding	Blinding was not relevant to our study because we do not compare the structures reported in the present study (on scarab beetle Chrysina gloriosa) to other structures found in other insect cuticles.

Reporting for specific materials, systems and methods

We require information from authors about some types of materials, experimental systems and methods used in many studies. Here, indicate whether each material, system or method listed is relevant to your study. If you are not sure if a list item applies to your research, read the appropriate section before selecting a response.

Materials & experimental systems

n/a	Involved in the study	
x		Antibodies
x		Eukaryotic cell lines
x		Palaeontology and archaeology
	x	Animals and other organisms
x		Human research participants
x		Clinical data
x		Dual use research of concern

Methods

n/a	Involved in the study	
x		ChIP-seq
x		Flow cytometry
x		MRI-based neuroimaging

Animals and other organisms

Policy information about studies involving animals; ARRIVE guidelines recommended for reporting animal research

Laboratory animals	The study did not involve living animals.
Wild animals	The study did not involve wild animals.
Field-collected samples	A male specimen of scarab beetle Chrysina gloriosa was collected at Madera Canyon (Santa Cruz County, Arizona, USA) in July 2014 by the insect seller. We bought the specimen to this insect seller and we received it as a dried sample. We have no more data on the parameters related to the collection of the specimen.
Ethics oversight	At our knowledge, no ethical approval or guidance was required to use this dried sample of scarab beetle Chrysina gloriosa.

Note that full information on the approval of the study protocol must also be provided in the manuscript.
